# Functional Outcomes of Treatment of Paediatric Supracondylar Fractures With Open or Closed Reduction and Crossed Kirschner Wire (K-wire) Fixation

**DOI:** 10.7759/cureus.105403

**Published:** 2026-03-17

**Authors:** Mahak Baid, Anirban Chatterjee, Shamik Hait, Jaya V Lal, Sunandan Datta

**Affiliations:** 1 Trauma and Orthopaedics, Aneurin Bevan University Health Board, Newport, GBR; 2 Trauma and Orthopaedics, Manipal Hospitals, Kolkata, IND; 3 Orthopaedics, Baksi Orthopaedic Trauma and Rehabilitation Centre, Kolkata, IND; 4 Trauma and Orthopaedics, Doncaster & Bassetlaw Teaching Hospitals NHS Foundation Trust, Doncaster, GBR

**Keywords:** elbow trauma, functional outcome after elbow fracture, k-wire fixation, mayo elbow performance score, supracondylar humeral fracture

## Abstract

Background: Supracondylar fractures of the humerus are a common type of elbow injury in children, and there is ongoing debate about the best method of surgical fixation.

Objectives: The aim of this study was to assess the functional outcome of paediatric supracondylar fractures managed using crossed Kirschner wire (K-wire) fixation.

Methods: This was a retrospective observational study. Thirty-one children (one to 14 years) with displaced supracondylar humerus fracture (Gartland types 2 and 3) treated with closed or open reduction and crossed K-wire fixation were included. Those with undisplaced fractures, flexion-type supracondylar fractures, open fractures, associated ipsilateral limb fractures, previous ipsilateral elbow injury, or neurovascular injury on presentation were excluded from the study. All patients were followed up for a period of one year, and functional outcome was evaluated using the Mayo Elbow Performance Score (MEPS).

Results: The mean age of the study population was 6.2 years (range: one to 14 years; SD: 3.6), with the majority of patients (51.6%) in the five- to nine-year age group. All fractures were of the extension type. Twenty-three patients (74.2%) sustained Gartland type III fractures, and eight patients (25.8%) had Gartland type II fractures. At the one-year follow-up, as evaluated by the MEPS, excellent functional outcomes were observed in 27 patients (87.1%), good outcomes in two patients (6.4%), and fair outcomes in two patients (6.4%). No individuals exhibited poor outcomes. Additionally, no cases of iatrogenic nerve injury, malunion, or residual deformity were noted at final follow-up.

Conclusion: Surgical fixation of supracondylar fractures with cross-pinning is an effective and safe modality for treating displaced paediatric supracondylar humerus fractures. When performed by an experienced surgeon using a mini-open medial approach, the risk of iatrogenic nerve injury is minimal. Large-scale prospective studies are required to further validate these findings.

## Introduction

Elbow fractures form a significant proportion of upper extremity fractures seen in children (8%-9%). Supracondylar fractures represent the most common paediatric elbow injury, comprising 55%-75% of the elbow fractures and accounting for 16.6% of overall childhood fractures [1]. These fractures are predominantly the extension type (95%) and are seen in five- to 10-year-old children [2]. The Gartland classification is the most widely used system and classifies the fracture depending on the extent of displacement. Type 1 fractures are undisplaced, type II fractures are displaced but with an intact posterior cortex, and type III fractures are displaced with no cortical contact [2,3]. This mechanism of injury for this fracture is typically due to a fall on an outstretched hand. These fractures are notorious for difficulty in achieving and maintaining reduction. Further, Leitch et al. added a type IV, which included fractures and multidirectional instability [4]. Additionally, 5%-30% of these fractures are associated with vascular and neural injury [5]. This association with neurovascular complications demands an aggressive approach and prudent management. Even an apparently uncomplicated fracture may lead to local swelling, deformity, and neurovascular complications if not treated properly; hence, these fractures demand an accurate neurovascular assessment and robust treatment plan. It’s essential to anticipate likely complications to diagnose and manage them properly.

The gold standard of management of displaced paediatric supracondylar fracture is closed reduction and fixation by percutaneous pinning under fluoroscopic guidance [6]. The technique for paediatric supracondylar fracture has been widely accepted and well-documented. There is controversy regarding the management of displaced supracondylar fractures, especially with regard to the ideal timing for surgery, methods for maintenance of reduction, and configuration of the pins used for fixation [7,8]. Bales et al., in their study, have shown that a delay in treating these fractures was not associated with a higher incidence of complications or need for open reduction, suggesting that delayed treatment of these fractures would not jeopardise the clinical outcome [9].

The commonly used pin configuration for management of these fractures is either cross-pinning or lateral pinning using two or three pins [10]. The technique of cross-pinning was first described by Swenson and has shown excellent results with low morbidity [11,12]. Although the cross-pin configuration is believed to be more stable mechanically to torsional forces across the fracture site as compared to lateral-only pins, there is a chance of iatrogenic ulnar nerve injury while inserting the medial pin [5,13,14]. Lateral-only pins are thought to provide inferior rotational stability to the fracture [15]. However, controversy still exists.

This retrospective study evaluates the clinical and functional outcomes of patients managed with closed or open reduction and cross-pinning, with particular focus on the incidence of iatrogenic ulnar nerve injury.

## Materials and methods

Study design

This was a retrospective observational study in which 31 children presenting with supracondylar humerus fracture were treated with open or closed reduction and cross-pinning in the orthopaedic department of Medica Superspecialty Hospital, a tertiary care hospital in Kolkata, India, over a two-year period between January 2013 and 2015, and fulfilled the inclusion and exclusion criteria.

Inclusion criteria

All individuals between one and 14 years of age who presented with displaced extension-type supracondylar fractures of the humerus and underwent closed or open reduction and percutaneous cross-pinning at the hospital between February 2010 and June 2016 were included in the study.

Exclusion criteria

Children with undisplaced fractures, flexion-type supracondylar fractures, open fractures, associated ipsilateral limb fractures (two), previous ipsilateral elbow injury, and children with neurovascular injury (five) were excluded from the study.

An initial group of 38 patients was selected for the study. Two individuals with concomitant both-bone forearm fractures, one with radial nerve palsy, three with median nerve involvement, and one with feeble pulses and inadequate perfusion were excluded from the study. A final group of 31 patients was taken following previous studies of a similar kind [16].

Operative protocol

Written informed consent was obtained from all parents. They were clinically and radiologically evaluated using routine anteroposterior and lateral view radiographs, and fracture configuration was classified based on Gartland classification [17]. The Gartland classification is a widely used, non-proprietary orthopaedic classification system described extensively in the literature and employed globally in clinical practice [17]. It is free to use and does not require licensing for research or publication purposes. An above-elbow slab was applied on admission, and all the children underwent fixation under general anaesthesia within 24 hours. Prophylactic antibiotics were administered in all cases before surgery.

The standard technique was followed for the reduction of the fracture. Reduction was achieved by manual inline traction with the elbow in extension, followed by flexion of the elbow with simultaneous application of pressure on the proximal surface of the olecranon. Image intensifier guidance was used to check the reduction. If the fracture could not be reduced to an acceptable position or if there was a neurovascular injury, an open reduction was performed. The elbow was kept hyper-flexed and in a position of pronation for inserting the lateral pin. The lateral pin was inserted first using a 1.5-mm percutaneous Kirschner wire (K-wire) placed from the lateral side of the elbow and engaging the medial cortex. The elbow was then extended to less than a 90° position, and a medial pin was inserted through a small incision after palpating and retracting the ulnar nerve posteriorly to avoid injury.

The pins were advanced just to perforate the opposite cortex for the best possible fixation. They were cut and bent 1 cm outside the skin. This would prevent migration and facilitate easy removal of the pins. Reduction, stability and pin placement were confirmed using an image intensifier before the application of a slab from the wrist to the axilla with the elbow maintained at 90° of flexion.

All patients were assessed post-operatively for the neurovascular status and discharged after one day. Active finger movement was advised post-operatively, and they were followed up at four weeks post-operatively for removal of the pins and back slab and to initiate range of motion exercises. Follow-up was arranged at six weeks, three months and one year, when X-rays were done along with assessment of the functional outcome using the Mayo Elbow Performance Score (MEPS) [18,19]. The MEPS is a validated, publicly available clinical assessment tool [18]. A standard analogue goniometer was used to assess the range of elbow motion. Additionally, any complications encountered were noted. The MEPS outcomes rated as excellent, good, or fair were regarded as satisfactory clinical results [19].

Statistical analysis

Statistical analysis was performed using IBM SPSS Statistics for Windows, version 20.0 (IBM Corp., Armonk, NY). Continuous variables, such as age, were expressed as mean ± standard deviation. Categorical variables, including age groups, sex, side of injury, and mechanism of injury, were expressed as numbers and percentages of patients. For age group comparisons, Pearson’s chi-square test was used to assess differences across categories. A one-sided binomial test was applied to evaluate sex distribution against the expected population sex ratio (female/male = 0.929). A p-value < 0.05 was considered statistically significant.

## Results

After inclusion and exclusion criteria, a final group of 31 individuals was chosen.

Demographics of the study group

Table 1 demonstrates the age demographics of the children undergoing surgery. The mean age at injury was 6.2±3.6 years (female: 5.4+/-2.91 years; male: 6.62+/-3.97 years), with the majority of the individuals in the five-to-nine-year age group, followed by 0 to four years of age, and the difference was statistically significant (chi-square test, P ≈0.00028). This statistically significant pattern reflects the greater vulnerability of younger children (<10 years) to these fractures.

**Table 1:** Age-wise distribution of children in the cohort df: degrees of freedom

Age (years)	Total	Percentage	(Observed – Expected)^2^ / Expected	P-value (Chi-square test)
0-4	11	35.5%	1.36	P=0.00028
5-9	16	51.6%	8.78	
10-14	3	9.6%	2.91	
15-18	1	3.2%	5.88	
			Chi-square value = 18.94 (df = 3)	

The observed sex distribution in our cohort (67.7% boys, 32.3% girls) did not differ significantly from the expected population ratio (female/male = 0.929; one-sided binomial test, p = 0.054), suggesting no statistically significant association between gender and incidence of supracondylar fracture in our cohort.

The left arm was injured in 22 (71%) of the children, and the right arm in nine (29%) of the children. All 31 children had sustained the extension type of supracondylar fracture. Twenty-three (74.2%) fractures were of Gartland type III, and eight (25.8%) were type II. Twenty-nine (93.5%) injuries occurred due to falls on outstretched hands, whereas two (6.5%) cases were due to road traffic accidents. Fracture was reduced by open reduction in six (19.3%) cases and by closed reduction in 25 (80.6%) cases. At final follow-up, no residual deformity was noted in any of the 31 children treated.

Functional outcomes based on MEPS

**Figure 1:**
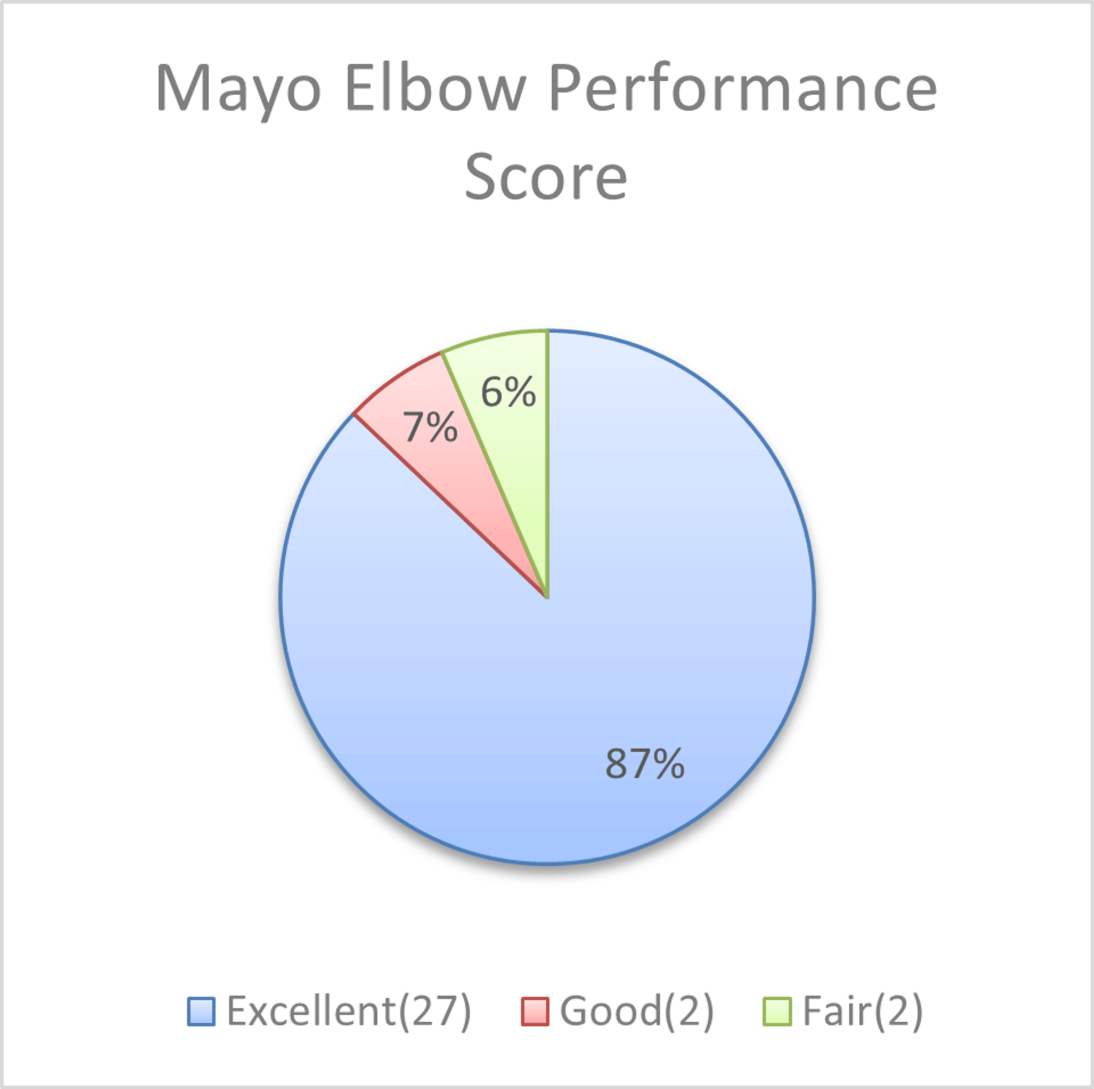
Functional outcomes based on the Mayo Elbow Performance Score Source: [18]

Figure 1 demonstrates the functional outcome, which was defined using MEPS [18]. Twenty-seven (87.1%) individuals achieved a score consistent with excellent clinical outcomes, two (6.45%) patients had a score consistent with a good functional outcome, and two (6.45%) patients had a score consistent with fair results. No individuals with poor outcomes were identified.

**Figure 2:**
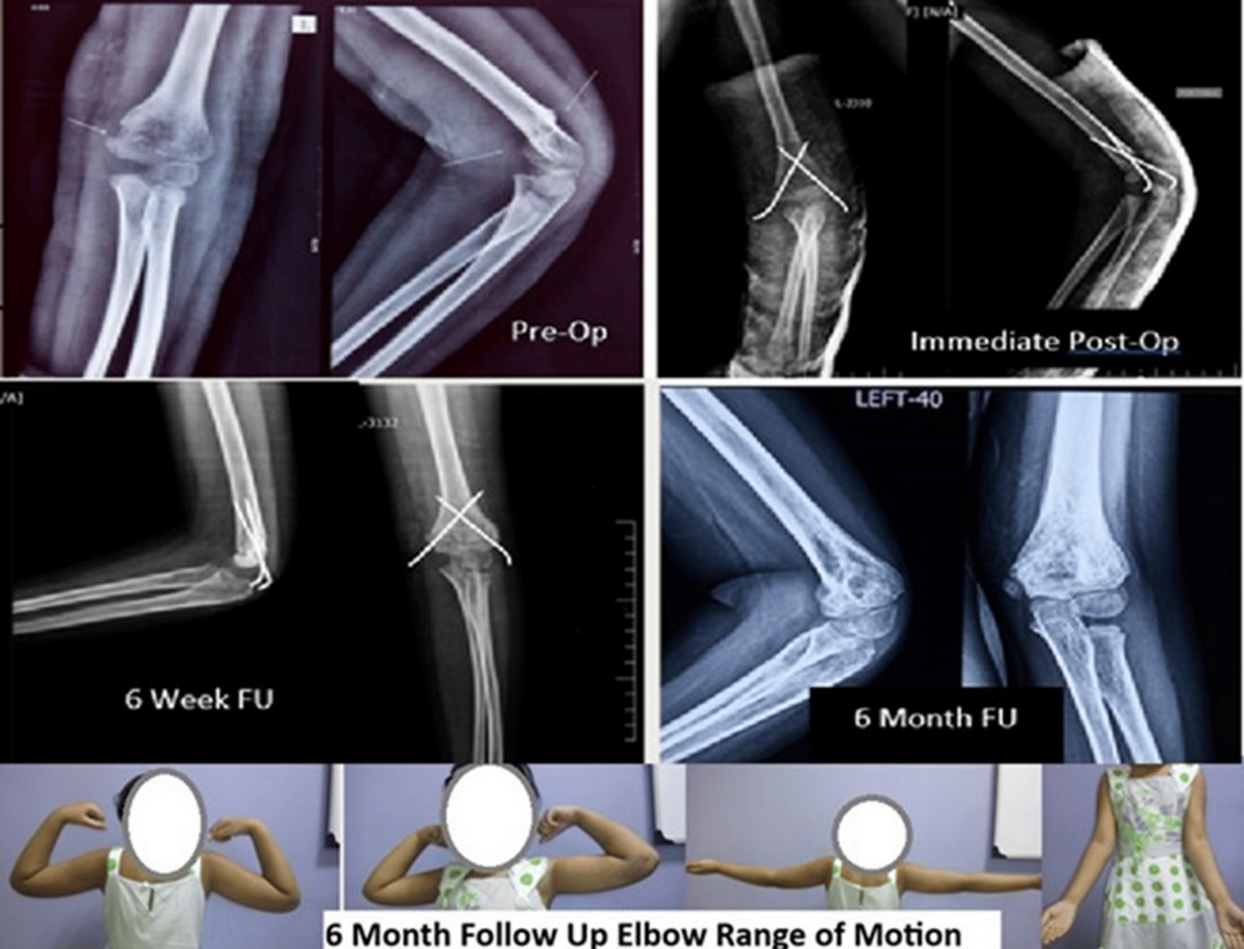
Functional outcomes at the one-year follow up (FU)

Figure 2 shows radiological images and clinical pictures at one-year follow-up. No cases of nerve injury were found in any of the 31 patients operated; however, there was one (3.2%) instance of pin tract infection in the open reduction group. None of the 31 patients treated had to undergo any reoperations.

## Discussion

The epidemiological data observed in our study are similar to earlier publications [20]. Supracondylar fractures continue to occur more often in boys, involve the left elbow (68% of boys in our study, with 61% of the injuries involving the left arm), and are most common when the child falls on an outstretched hand. These trends closely mirror recent epidemiological studies as well [21].

A reassuring finding from our study was the absence of post-operative nerve injury among the patients in our cohort. In the past, studies have indicated that nerve injury occurs in roughly 12%-20% of cases [22,23]. However, recent literature has reported that these complications have become less frequent, owing to better understanding and enhanced procedural technique. According to a meta-analysis conducted by Zhao et al., the risk of iatrogenic nerve injury in injuries treated with the crossed pinning technique via a mini-open medial incision was the same as that of injuries treated with lateral pinning [24]. Our study echoed these findings, reinforcing the idea that careful tissue handling during medial pinning (via mini-open approach) is key to preventing iatrogenic nerve injuries.

Cross-pinning continues to offer excellent biomechanical stability when compared to lateral pin constructs, particularly with respect to rotational and torsional stability [24,25]. Our clinical findings are consistent with this evidence: 27 of the 31 patients achieved excellent outcomes according to the MEPS, while two demonstrated good results and two achieved fair functional recovery. Our series demonstrated a low frequency of complications, with only a single case of pin tract infection, which resolved uneventfully with oral antibiotics. Importantly, no instances of compartment syndrome, deep infection, osteomyelitis, or loss of reduction were recorded. These outcomes support the current literature indicating that crossed K-wire fixation is a reliable and effective treatment modality for Gartland type II and III supracondylar fractures when executed with appropriate technique.

The correlation between surgical timing and the likelihood of requiring open reduction remains a subject of ongoing debate. Walmsley et al., in their review of 171 Gartland type III fractures, observed a significantly increased rate of open reduction when operative management was delayed beyond eight hours (33.3% vs. 11.2%) [26]. In contrast, Alshayan et al. reported that delayed surgical intervention did not adversely affect complication rates; however, they noted that procedures performed outside regular operating hours were associated with a higher incidence of neurovascular injuries [27]. Similar results were obtained by Abrahim et al., who noted a higher occurrence of complications when surgeries were conducted out of working hours [28]. Considering these observations, our view is that, in the absence of neurovascular compromise, operative treatment of supracondylar humerus fracture can be deferred until the next day, thus ensuring that surgery is performed under optimal conditions by an experienced orthopaedic surgeon.

Our study has certain limitations that must be addressed. The relatively limited sample size restricts the broader applicability of our results. In addition, although a one-year follow-up period is sufficient for assessing early functional outcomes, it does not rule out late complications such as a malunion and cubitus varus deformity. Despite these limitations, our results offer insight into the safety and efficacy of crossed K-wire fixation for paediatric supracondylar humerus fractures. Notably, the absence of iatrogenic nerve injury in our cohort highlights the significance of refined surgical technique, particularly the use of a mini-open approach for medial pin. Further validation of these findings requires large-scale, prospective studies with extended follow-up periods.

## Conclusions

Crossed K-wire fixation remains a stable treatment option for displaced Gartland II and III supracondylar humerus fractures in children. It provides excellent fixation with minimal risk of iatrogenic nerve injury when performed by an experienced surgeon via a mini-open medial approach. Further research involving bigger patient groups and longer follow-up periods is necessary to validate these results.
